# The Predictive Power of the Cystatin C‐Creatinine Score in Assessing Frailty

**DOI:** 10.1002/jcsm.70040

**Published:** 2025-08-15

**Authors:** Li Deng, Xin Zheng, Yue Chen, Chenan Liu, Jinyu Shi, Zhaoting Bu, Xiaoyue Liu, Hong Zhao, Shuqun Li, Bing Yin, Siyu Xing, Hanping Shi

**Affiliations:** ^1^ Department of Gastrointestinal Surgery, Department of Clinical Nutrition, Beijing Shijitan Hospital Capital Medical University Beijing China; ^2^ National Clinical Research Center for Geriatric Diseases Xuanwu Hospital, Capital Medical University Beijing China; ^3^ Key Laboratory of Cancer FSMP for State Market Regulation Beijing China; ^4^ Laboratory for Clinical Medicine Capital Medical University Beijing China; ^5^ The Second Affiliated Hospital and Yuying Children's Hospital of Wenzhou Medical University Wenzhou China; ^6^ Department of Biology, Faculty of Science The University of Hong Kong Hong Kong China

**Keywords:** ageing, creatinine, cystatin C, frailty, mortality

## Abstract

**Background:**

As the global population ages, identifying reliable biomarkers to predict frailty and mortality is critical for early intervention. This study aims to construct a valuable biomarker and evaluate its predictive performance in assessing frailty and all‐cause mortality.

**Methods:**

Data from 3613 participants in the Health and Retirement Study (HRS) were used to construct the nomogram and main analysis, whereas data from the National Health and Nutrition Examination Survey were used to validate the robustness of the model. LASSO regression identified key biomarkers, and a nomogram was used to construct the score. The frailty index (FI) and all‐cause mortality were used as the outcomes, and the score's predictive ability was evaluated using ROC curves, C‐index and decision curve analysis. Subgroup analyses were conducted to assess the score's consistency across age, sex and clinical conditions.

**Results:**

Sixteen haematological markers were selected through LASSO regression. The nomogram demonstrated that a scoring model based on cystatin C and creatinine can achieve optimal predictive performance. The Cystatin C‐Creatinine Score demonstrated strong predictive power for frailty (AUC = 0.687) and all‐cause mortality (AUC = 0.733). Logistic regression analysis showed a significant association between higher Cystatin C‐Creatinine Scores and increased frailty risk, with participants in the high‐risk group having an OR of 1.48 (95% CI: 1.35–1.62, *p* < 0.001) compared to the low‐risk group. Cox proportional hazards models also indicated that higher scores were associated with increased mortality risk (HR = 3.34, 95% CI: 1.75–6.38, *p* < 0.001 for the high‐risk group). In the validation set, the AUC values of the Cystatin C‐Creatinine Score for predicting frailty and all‐cause mortality reached 0.701 and 0.713, respectively.

**Conclusion:**

Our findings support the use of the Cystatin C‐Creatinine Score as a practical and effective tool for identifying individuals at higher risk of frailty and mortality.

## 1 Introduction

Frailty is a clinical syndrome characterized by a heightened vulnerability to stressors resulting from cumulative declines in multiple physiological systems, leading to increased risks of disability, hospitalization and mortality [1, 2]. As the global population continues to age, identifying reliable biomarkers for predicting frailty and mortality is crucial for early intervention [2, 3]. Frailty and mortality are key manifestations of functional ageing, which refers to the decline in physical and cognitive functions that impair an individual's ability to perform daily activities [4, 5]. Unlike biological ageing, which is often assessed through cellular and molecular changes, functional ageing more directly reflects a person's vulnerability to adverse health outcomes [5, 6].

Although physical performance measures and self‐reported questionnaires provide valuable insights, the integration of biological markers offers an objective, quantifiable method for assessing physiological decline, potentially enhancing early detection and intervention efforts [7]. Among the biological markers that have gained attention, epigenetic clocks estimate biological age based on DNA methylation patterns and have shown promise in predicting age‐related outcomes [6, 8]. However, their ability to reflect functional ageing—particularly frailty and mortality risk—remains uncertain. Though epigenetic clocks can provide insight into biological age, their limitations in capturing functional decline have become increasingly evident.

Given the uncertainty surrounding epigenetic clocks in predicting functional ageing, the study turned our focus to haematological biomarkers, which directly reflect key physiological processes such as kidney function and muscle mass [7, 9]. These markers may provide a more accurate assessment of both functional and biological ageing. Cystatin C and creatinine, in particular, have been widely studied as indicators of renal function and muscle mass, respectively [9, 10, 11, 12]. Their combined use offers a practical approach to assessing frailty and mortality risk, potentially filling the gap left by epigenetic clocks.

Additionally, by operationalizing our findings into a user‐friendly online calculator, this study aims to bridge the gap between research and clinical practice, allowing clinicians to easily assess frailty risk using accessible biomarkers. This practical tool could enhance early detection, allowing for timely interventions to mitigate frailty and its associated risks.

## 2 Methods

### 2.1 Participants

The Health and Retirement Study (HRS) is a nationally representative longitudinal panel study conducted by the University of Michigan [13]. Initiated in 1992, it follows up every 2 years and primarily investigates the health, work and retirement, social relationships and economic status of US adults aged 50 and older, along with their spouses. The HRS uses a multi‐stage area probability design to sample participants from multiple age cohorts, covering a broad range of ageing‐related topics, making it the most comprehensive study on ageing in the United States. The collection and production of HRS data strictly adhere to the requirements of the University of Michigan's Institutional Review Board, and all participants provided informed consent, ensuring data quality and ethical compliance.

**FIGURE 1:**
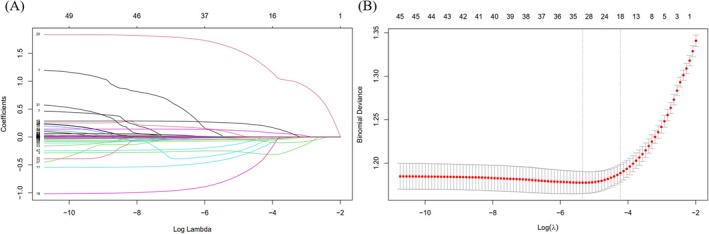
LASSO regression for variable selection. (A) Coefficient paths across different lambda values. (B) Cross‐validation for optimal lambda selection.

This study utilized the 2016 HRS core data and the Venous Blood Study (VBS) data, with follow‐up extending through 2020. The initial sample consisted of 4018 participants with complete data for 13 epigenetic age clocks. After excluding 289 participants due to missing components of the frailty index (FI) and another 289 participants due to missing haematological data, a final total of 3613 participants was retained for analysis. A detailed flow chart of the participant selection process is presented in Figure 1.

To verify the robustness of the results, we also included the population data from the National Health and Nutrition Examination Survey (NHANES) from 1999 to 2002. This is because during these years, the levels of cystatin C were measured for the majority of the participants. After excluding the participants who did not undergo the measurement of cystatin C, creatinine, prognosis assessment, and those whose data were not available for calculating the frailty score, a total of 3639 participants were included in the validation cohort (Table 1).

**TABLE 1:** Baseline characteristics.

Characteristics	Overall	Frailty		*p*
		No	Yes	
*N*	3613	2176	1437	
Age (mean [SD])	69.52 (9.55)	68.22 (9.03)	71.50 (9.99)	< 0.001
Sex (%)				
Male	1443 (40.42)	930 (43.06)	513 (36.38)	< 0.001
Female	2127 (59.58)	1230 (56.94)	897 (63.62)	
Marital status (%)				
No	1491 (41.76)	766 (35.46)	725 (51.42)	< 0.001
Yes	2079 (58.24)	1394 (64.54)	685 (48.58)	
Education (mean [SD])	12.91 (3.16)	13.40 (2.96)	12.16 (3.31)	< 0.001
SRH (median [IQR])	3.00 [2.00, 4.00]	2.00 [2.00, 3.00]	4.00 [3.00, 4.00]	< 0.001
Hypertension (%)				
No	1323 (37.06)	1042 (48.24)	281 (19.93)	< 0.001
Yes	2247 (62.94)	1118 (51.76)	1129 (80.07)	
Diabetes (%)				
No	2617 (73.31)	1798 (83.24)	819 (58.09)	< 0.001
Yes	953 (26.69)	362 (16.76)	591 (41.91)	
Cancer (%)				
No	2985 (83.61)	1902 (88.06)	1083 (76.81)	< 0.001
Yes	585 (16.39)	258 (11.94)	327 (23.19)	
Alcohol consumption (%)				
No	2018 (56.53)	1357 (62.82)	661 (46.88)	< 0.001
Yes	1552 (43.47)	803 (37.18)	749 (53.12)	
Smoking status (%)				
No	3186 (89.24)	1955 (90.51)	1231 (87.30)	0.003
Yes	384 (10.76)	205 (9.49)	179 (12.70)	
Status (%)				
Alive	3263 (91.40)	2063 (95.51)	1200 (85.11)	< 0.001
Dead	307 (8.60)	97 (4.49)	210 (14.89)	
Depression (median [IQR])	2.00 [2.00, 4.00]	2.00 [2.00, 3.00]	3.00 [2.00, 5.00]	< 0.001
Cognition (median [IQR])	14.00 [11.00, 17.00]	15.00 [11.00, 18.00]	13.00 [10.00, 16.00]	< 0.001
HORVATH_DNAMAGE (mean [SD])	65.71 (9.52)	64.66 (9.04)	67.31 (9.99)	< 0.001
HANNUM_DNAMAGE (mean [SD])	54.57 (9.14)	53.32 (8.83)	56.49 (9.28)	< 0.001
LEVINE_DNAMAGE (mean [SD])	57.40 (10.00)	55.77 (9.52)	59.91 (10.21)	< 0.001
HORVATHSKIN_DNAMAGE (mean [SD])	69.62 (8.77)	68.44 (8.45)	71.42 (8.94)	< 0.001
LIN_DNAMAGE (mean [SD])	58.36 (11.01)	57.11 (10.68)	60.29 (11.24)	< 0.001
WEIDNER_DNAMAGE (mean [SD])	67.27 (11.63)	66.47 (11.20)	68.49 (12.15)	< 0.001
VIDALBRALO_DNAMAGE (mean [SD])	63.71 (6.10)	62.99 (5.75)	64.82 (6.44)	< 0.001
YANG_DNAMAGE (median [IQR])	0.07 [0.06, 0.08]	0.06 [0.06, 0.08]	0.07 [0.06, 0.08]	< 0.001
ZHANG_DNAMAGE (median [IQR])	−1.12 [−1.42, −0.80]	−1.19 [−1.48, −0.88]	−1.01 [−1.32, −0.69]	< 0.001
BOCKLANDT_DNAMAGE (median [IQR])	0.39 [0.34, 0.44]	0.39 [0.35, 0.44]	0.38 [0.33, 0.43]	< 0.001
GARAGNANI_DNAMAGE (median [IQR])	0.71 [0.67, 0.76]	0.70 [0.66, 0.75]	0.72 [0.68, 0.78]	< 0.001
DNAMGRIMAGE (mean [SD])	68.00 (8.59)	66.38 (8.23)	70.47 (8.54)	< 0.001
MPOA (median [IQR])	1.07 [1.01, 1.13]	1.06 [1.00, 1.12]	1.08 [1.03, 1.14]	< 0.001
Creatine (median [IQR])	0.87 [0.73, 1.04]	0.86 [0.73, 1.01]	0.88 [0.74, 1.10]	< 0.001
Cystatin C (median [IQR])	1.07 [0.91, 1.30]	1.02 [0.88, 1.19]	1.19 [0.99, 1.44]	< 0.001

*Note:* Continuous variables are presented as mean ± standard deviation, and categorical variables are presented as numbers (percentages). Differences in baseline characteristics were compared using the χ_2_ test for categorical variables and the two‐sample *t*‐test for continuous variables.

### 2.2 Evaluation of Frailty

In this study, the FI was used to assess participants' frailty levels [14, 15]. The FI was determined by calculating the cumulative health deficits associated with ageing. It was based on 30 health deficit items, including chronic diseases, self‐reported health status, functional limitations, depressive symptoms and cognitive function (Methods S1). Each health deficit item was assigned a score based on its severity. Most items were scored as 0 if the deficit was absent and 1 if it was present. Some items, like self‐reported vision, hearing, health status and cognitive function, were scored on a scale from 0 to 1, with higher values indicating more severe deficits. The final FI score was calculated by summing all item scores, dividing by the total number of items and multiplying by 100. This produced a continuous variable ranging from 0 to 100. In this study, frailty status was defined as a binary variable based on the FI score. Participants with an FI score of ≥ 25 were classified as frail, whereas those with a score of < 25 were classified as non‐frail.

### 2.3 Evaluation of All‐Cause Mortality

The HRS respondent records were matched to the National Death Index (NDI), with data coverage up to 2013 [16, 17]. The NDI is a comprehensive mortality database managed by the National Center for Health Statistics (NCHS). Through NDI matching, accurate mortality information for participants can be obtained, including the date of death and cause of death classification. For data after 2013, due to delays in NDI data, the study relied on proxy reports of the participants' mortality provided by family members. These reports were regularly updated through 2019. Survival time was calculated based on the interval from the baseline interview month (2016) to the month of the last interview or the month of death.

### 2.4 Evaluation of Epigenetic Age

In this study, the assessment of epigenetic age was based on DNA methylation data from the HRS [6]. This study utilized 13 epigenetic clocks to estimate participants' biological age, which predict age‐related changes by analysing DNA methylation patterns at specific genomic sites. These 13 epigenetic clocks include Horvath's Clock, Hannum's Clock, PhenoAge and GrimAge, among others, each optimized for different biological and clinical endpoints. For instance, Horvath's Clock is a universal clock suitable for various tissues and cell types, whereas GrimAge incorporates clinical risk factors to predict lifespan and health risks. Additionally, PhenoAge incorporates clinical biomarkers to more accurately reflect an individual's health status. Among these, 11 clocks were constructed by Morgan Levine (Yale University) and the HRS staff to ensure reliability. GrimAge, the 12th clock, was developed by HRS staff member Jonah Fisher with the assistance of Steve Horvath. The 13th clock, DunedinPoAm38, was estimated by Thalida Arpawong (University of Southern California) with the assistance of Karen Sugden (Duke University). Detailed information on the structure of these 13 epigenetic clocks can be found in other specialized studies [6].

### 2.5 Construction of Cystatin C‐Creatinine Score

This study firstly extracted over 40 haematological biomarkers from the 2016 VBS dataset and used frailty as the outcome variable. Then, the Least Absolute Shrinkage and Selection Operator (LASSO) regression model was applied to screen these variables. LASSO regression is a statistical method used for high‐dimensional data analysis that introduces an L1 regularization constraint, allowing the regression model to automatically select the most influential variables for predicting frailty. After identifying key haematological biomarkers through LASSO regression, the study constructed a nomogram based on these variables. To ensure that the chosen biomarkers are not overly specific to the HRS cohort, 10‐fold cross‐validation was considered to be used for confirming the robustness of the selected variables. The nomogram translates the results of the regression model into an easily interpretable scoring system for predicting individual frailty risk. In the nomogram, each variable is assigned a weight based on its regression coefficient, with higher scores indicating a greater contribution of that variable to frailty. Finally, the cystatin C‐creatinine score for each participant was calculated based on the constructed nomogram (Methods S2). To facilitate the calculation, we designed a web‐based calculator (https://frailtycal.shinyapps.io/shiny_app/).

### 2.6 Covariates

In our study models, several covariates were included based on a literature review and prior research that have demonstrated potential associations with epigenetic age and frailty. Specifically, the covariates included age, gender, education level, marital status, smoking, estimated glomerular filtration rate (eGFR) and alcohol consumption. Age was calculated based on the participant's actual age at the time of the interview. Gender was treated as a binary variable, classified as either male or female. Education level was treated as a continuous variable, reflecting the number of years of education completed by the participants. Smoking and alcohol consumption were categorized as current smoker/drinker or non‐smoker/non‐drinker. Additionally, in the models where mortality was the outcome, this study included a history of diabetes, hypertension, heart disease and cancer as covariates. The presence of these conditions was determined through self‐reports by the participants. These covariates were included in our models to control for potential confounding factors, thereby allowing for a more accurate assessment of the relationship between haematological biomarkers and both frailty and physiological ageing (all‐cause mortality).

### 2.7 Statistical Analysis

In the statistical analysis section, baseline characteristics between frail and non‐frail participants were first compared. Independent samples *t*‐tests or Mann–Whitney *U* tests (for non‐normally distributed data) were used for continuous variables, whereas chi‐square tests or Fisher's exact tests (for cases with small expected frequencies) were employed for categorical variables. Next, the predictive performance of the constructed frailty score and the 13 epigenetic age markers for frailty was evaluated. Receiver operating characteristic (ROC) curves and c‐index were used to assess the discriminatory ability of the models, and calibration curves were plotted to evaluate the accuracy of the predictions. Additionally, decision curve analysis (DCA) was conducted to assess the clinical utility of the models at different thresholds. For the determination of the cut‐off value of the Cystatin C‐Creatinine Score, we used the ‘maxstat’ package to carry out the operation (Methods S3). Further analysis involved using logistic regression models to calculate the odds ratio (OR) of the frailty score, quantifying its association with frailty status. Moreover, timeROC curves comparing the frailty score with the 13 epigenetic age markers for predicting mortality were analysed, and Cox proportional hazards models were used to calculate hazard ratios (HR) to evaluate the predictive power of these scores for mortality. Finally, to validate the fairness of the frailty score across different subgroups, stratified analyses were conducted, assessing the consistency and applicability of the score across various subgroups such as age, gender, and race. All analyses were performed using R 4.4.0. A two‐sided *p* < 0.05 was considered statistically significant.

## 3 Results

### 3.1 Baseline Characteristics

The study included 3613 participants, with 1437 (39.78%) classified as frail. The frail group had a higher average age (71.50 years vs. 68.22 years, *p* < 0.001), a larger proportion of females (63.62% vs. 56.94%, *p* < 0.001), a higher percentage of unmarried individuals (51.42% vs. 35.46%, *p* < 0.001) and fewer years of education (12.16 years vs. 13.40 years, *p* < 0.001). The frail group reported worse self‐rated health (median = 4 vs. 2, *p* < 0.001) and had significantly higher rates of hypertension and diabetes (80.07% and 41.91% vs. 48.24% and 16.76%, *p* < 0.001). In terms of lifestyle factors, the frail group had higher proportions of smokers (12.70% vs. 9.49%, *p* < 0.001) and alcohol consumers (53.12% vs. 37.18%, *p* < 0.001). Additionally, the frail group showed a higher mortality rate (14.89% vs. 4.49%, *p* < 0.001) and greater depression scores (median = 3 vs. 2, *p* < 0.001). Overall, frail individuals were at a disadvantage regarding age, health status and lifestyle factors.

### 3.2 Haematological Biomarker Selection and Nomogram Construction

**FIGURE 2:**
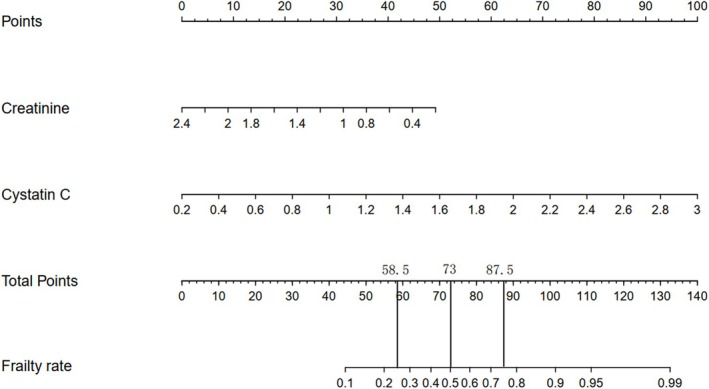
Construction of a nomogram for predicting frailty.

LASSO regression was used to identify key haematological biomarkers associated with frailty. Figure 1A displays the coefficient paths of various biomarkers as a function of lambda, whereas Figure 1B shows the cross‐validation used to select the optimal lambda value. From this analysis, cystatin C and creatinine emerged as the two most significant biomarkers for predicting frailty (Table S1). Following this, nomograms were constructed using different sets of biomarkers selected by LASSO, specifically the top 2, top 4, top 5, and top 16 biomarkers (Figures 2 and S2). These nomograms were used to predict frailty, and their predictive performance was evaluated using ROC curves. Figure S2 illustrates the constructed nomograms and the comparison of their ROC curves. As shown in Figure S2, the nomogram with only creatinine and cystatin C (AUC = 0.696) provided reasonable predictive accuracy. Adding more biomarkers improved the model's performance, with the nomogram using the top 16 biomarkers achieving the highest AUC (0.738). However, the simpler model with creatinine and cystatin C already offered strong predictive capability, suggesting its utility in clinical settings. The results of the internal 10‐fold cross‐validation also indicate that the variables selected by LASSO regression have high accuracy (Figure S3). Compared with the sole use of traditional renal function indicators (such as eGFR or creatinine), this nomogram can more effectively predict the frailty risk of participants (Figure S4).

### 3.3 Nomogram Construction and Evaluation

**FIGURE 3:**
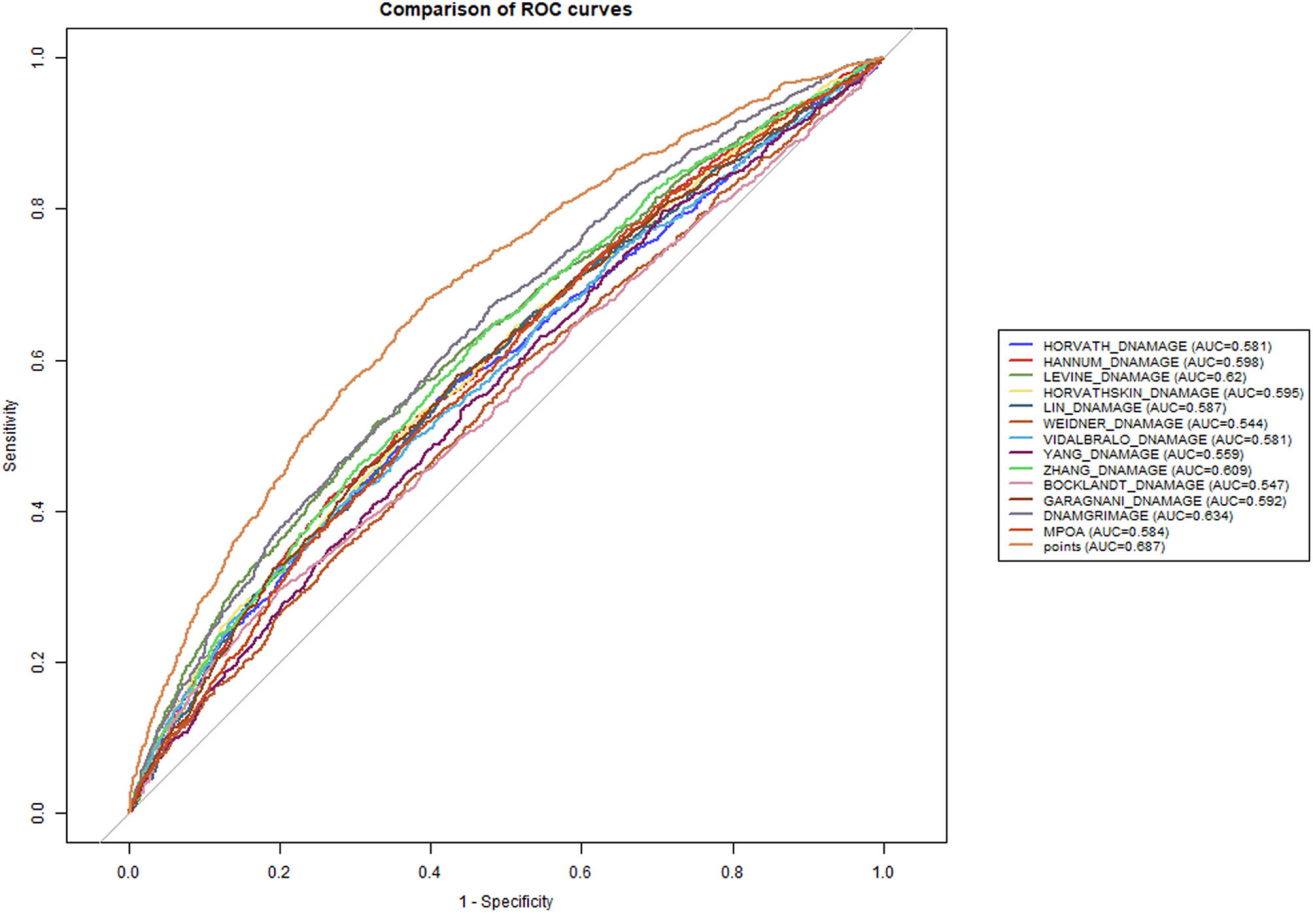
Comparison of ROC curves for predicting frailty across Cystatin C‐Creatinine Score and epigenetic clocks.

**FIGURE 4:**
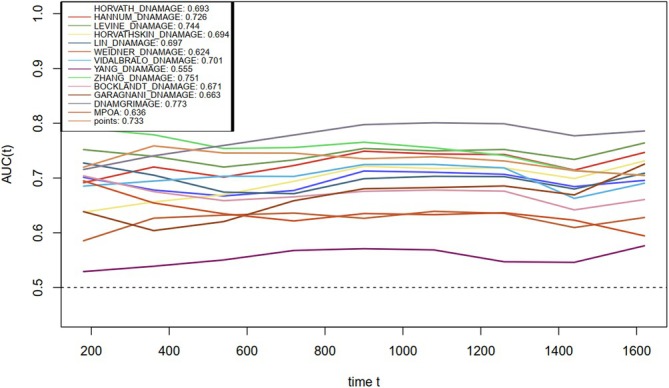
Time‐dependent ROC curves comparing Cystatin C‐Creatinine Score and epigenetic clocks for predicting mortality.

Using the nomogram, the Cystatin C‐Creatinine Score was calculated, and its relationship with frailty and all‐cause mortality was further evaluated. As shown in Figure S5A,B, the Cystatin C‐Creatinine Score was positively associated with both frailty and all‐cause mortality rates. As the score increased, the risk of frailty and death also rose. Figure 3 illustrates the comparison of ROC curves for the Cystatin C‐Creatinine Score and the 13 epigenetic clocks in predicting frailty. The results demonstrate that the Cystatin C‐Creatinine Score achieved an AUC of 0.687, outperforming many of the epigenetic clocks. Figure 4 further shows the time‐dependent ROC curve comparison between the Cystatin C‐Creatinine Score and the 13 epigenetic clocks in predicting all‐cause mortality. As shown in the figure, the Cystatin C‐Creatinine Score maintained a stable AUC value of 0.733 over time and demonstrated consistent predictive power at different time points. Although some epigenetic clocks, such as DNAmGrimAge (AUC = 0.781) and Hannum DNAmAge (AUC = 0.728), performed well, the Cystatin C‐Creatinine Score showed comparable or superior predictive ability compared to these clocks. Additionally, Figure S6 illustrates the linear correlation between different epigenetic clocks, the Cystatin C‐Creatinine Score and frailty. The Cystatin C‐Creatinine Score showed the strongest positive correlation with frailty (correlation coefficient = 0.38), outperforming all other epigenetic clocks (Figure S7).

### 3.4 Association of Cystatin C‐Creatinine Score With Frailty and All‐Cause Mortality

We first explored the relationship between the Cystatin C‐Creatinine Score and systemic inflammation (Figure S8). The results showed that the Cystatin C‐Creatinine Score was positively correlated with an increase in the body's inflammatory level. The relationship between the Cystatin C‐Creatinine Score and frailty was evaluated using multivariate logistic regression models, as shown in Table S2. When treated as a continuous variable, the Cystatin C‐Creatinine Score was significantly associated with an increased risk of frailty (OR = 1.01, 95% CI: 1.01–1.01, *p* < 0.001). Using a cut‐off value of 67.7, participants with a score ≥ 67.7 had a 1.20‐fold higher risk of frailty compared to those with a score < 67.7 (OR = 1.20, 95% CI: 1.16–1.24, *p* < 0.001). When analysed by quartile, the highest quartile had a 1.33‐fold higher frailty risk compared to the lowest quartile (OR = 1.33, 95% CI: 1.27–1.40, *p* < 0.001). Similarly, the association between the Cystatin C‐Creatinine Score and all‐cause mortality was evaluated using Cox proportional hazards models, as shown in Table S3. As a continuous variable, the score was significantly associated with an increased risk of mortality (HR = 1.03, 95% CI: 1.02–1.04, *p* < 0.001). Participants with a score ≥ 67.7 had a 2.06‐fold higher risk of all‐cause mortality compared to those with a score < 67.7 (HR = 2.06, 95% CI: 1.56–2.71, *p* < 0.001). The highest quartile (Q4, ≥ 71.56) had a 2.64‐fold increased risk of mortality compared to the lowest quartile (Q1, < 60.85) (HR = 2.64, 95% CI: 1.69–4.11, *p* < 0.001).

Table 2 categorizes participants into four risk groups based on frailty incidence predicted by the Cystatin C‐Creatinine Score from the nomogram: low risk (< 58.5, frailty rate < 0.25), low‐medium risk (58.5–73, frailty rate 0.25–0.5), medium‐high risk (73–87.5, frailty rate 0.5–0.75) and high risk (> 87.5, frailty rate > 0.75). Compared to the low‐risk group, the low‐medium risk group had a 1.12‐fold higher risk of frailty (OR = 1.12, 95% CI: 1.07–1.18, *p* < 0.001), the medium‐high risk group had a 1.31‐fold higher risk (OR = 1.31, 95% CI: 1.23–1.39, *p* < 0.001), and the high‐risk group had a 1.48‐fold increased risk (OR = 1.48, 95% CI: 1.35–1.62, *p* < 0.001). For all‐cause mortality, participants in the medium‐high risk group had a 2.24‐fold increased risk (HR = 2.24, 95% CI: 1.25–4.00, *p* = 0.007), whereas those in the high‐risk group had a 3.34‐fold higher risk (HR = 3.34, 95% CI: 1.75–6.38, *p* < 0.001), compared to the low‐risk group.

**TABLE 2:** The relationship of Cystatin C‐Creatinine Score (categorized by frailty risk as low, low‐medium, medium‐high and high risk) with frailty and all‐cause mortality.

Frailty	Frailty/participants	Model 1	*p*	Model 2	*p*	Model 3	*p*
Low risk (~58.5)	98/522	Ref	Ref	Ref	Ref	Ref	Ref
Low‐medium risk (58.5–73)	843/2303	1.20 (1.14, 1.25)	< 0.001	1.15 (1.10, 1.20)	< 0.001	1.12 (1.07, 1.18)	< 0.001
Medium‐high risk (73–87.5)	365/610	1.51 (1.43, 1.59)	< 0.001	1.38 (1.30, 1.46)	< 0.001	1.31 (1.23, 1.39)	< 0.001
High risk (87.5–)	104/135	1.79 (1.64, 1.96)	< 0.001	1.58 (1.44, 1.74)	< 0.001	1.48 (1.35, 1.62)	< 0.001
*p* for trend			< 0.001		< 0.001		< 0.001

All‐cause mortality	Death/participants	Model 1	*p*	Model 2	*p*	Model 3	*p*
Low risk (~58.5)	15/522	Ref	Ref	Ref	Ref	Ref	Ref
Low‐medium risk (58.5–73)	147/2303	2.21 (1.30, 3.76)	0.003	1.52 (0.89, 2.62)	0.128	1.37 (0.79, 2.36)	0.261
Medium‐high risk (73–87.5)	104/610	6.28 (3.66, 10.79)	< 0.001	2.71 (1.52, 4.82)	0.001	2.24 (1.25, 4.00)	0.007
High risk (87.5–)	41/135	12.36 (6.84, 22.33)	< 0.001	4.25 (2.25, 8.03)	< 0.001	3.34 (1.75, 6.38)	< 0.001
*p* for trend			< 0.001		< 0.001		< 0.001

*Note:* Model 1: no adjusted. Model 2: adjusted for age, sex, marital status and education level. Model 3: adjusted for age, sex, marital status, education level, smoking status, alcohol consumption, albumin, bilirubin, alanine aminotransferase and alkaline phosphatase.

To further evaluate the effectiveness of the Cystatin C‐Creatinine Score, we conducted a validation in the NHANES cohort. The results showed that the area under the curve (AUC) value of the Cystatin C‐Creatinine Score for predicting the frailty status was 0.701, and the AUC value for predicting the all‐cause mortality of the participants in terms of prognosis was 0.713 (Figure S9).

### 3.5 Comparison of Predictive Performance Across Models

Table S4 shows the C‐index for predicting frailty, with the Cystatin C‐Creatinine Score achieving a C‐index of 0.717, comparable to epigenetic clocks like DNAmGrimAge (C‐index = 0.781) and Hannum DNAmAge (C‐index = 0.728). Figures S10 and S11 show that the Cystatin C‐Creatinine Score consistently predicts frailty and all‐cause mortality across different subgroups. The score performed similarly in participants of different ages, sexes, marital statuses, education levels and those with or without chronic conditions such as hypertension, diabetes and cancer.

## 4 Discussion

This study identified and validated the Cystatin C‐Creatinine Score as a robust predictor of both frailty and all‐cause mortality. The score demonstrated strong predictive performance, with an AUC of 0.687 for frailty and 0.733 for all‐cause mortality, making it a valuable tool in assessing functional ageing. Importantly, the Cystatin C‐Creatinine Score outperformed several widely recognized epigenetic clocks, such as DNAmGrimAge and Hannum DNAmAge, particularly in predicting frailty. This highlights the unique advantage of using blood‐based biomarkers, like cystatin C and creatinine, which are directly reflective of physiological functions closely related to frailty and mortality. Furthermore, subgroup analyses showed that the Cystatin C‐Creatinine Score remained consistently accurate across different subgroups. This suggests that the score is broadly applicable in various demographic and clinical populations.

Our study's findings align with previous research demonstrating the predictive value of the Cystatin C‐Creatinine ratio in relation to frailty and mortality. Several studies have shown that the Cystatin C‐Creatinine ratio serves as an effective biomarker for muscle mass and sarcopenia, reflecting overall health deterioration in ageing populations [9, 18, 19, 20, 21, 22]. For instance, Zhang et al. found that lower levels of the creatinine/cystatin C ratio (Cr/CysC) are independently associated with higher all‐cause mortality in adults over 80 [18]. Similarly, Kitago et al. demonstrated a cross‐sectional and longitudinal association between the creatinine‐to‐cystatin C ratio and sarcopenia parameters such as handgrip strength and skeletal muscle mass, further supporting the use of this ratio as a screening tool for sarcopenia [19]. In our study, the frailty risk prediction using the Cystatin C‐Creatinine Score demonstrated robust associations with frailty rates and mortality across different subgroups, as shown in Figures S6 and S7. This is consistent with findings from Kitago et al., where a significant dose–response relationship between the score and muscle strength decline was reported over a 6‐year follow‐up. Additionally, our findings are corroborated by the work of Osaka et al., which highlighted the role of the Cystatin C‐Creatinine ratio as a surrogate marker of sarcopenia, particularly in diabetic patients [23]. However, we compared the ROC curves of the Cystatin C‐Creatinine Score and the creatinine‐to‐cystatin C ratio for predicting frailty, and the results showed that the Cystatin C‐Creatinine Score had a superior AUC value (Figure S2D). The Cystatin C‐Creatinine Score offers an accessible and cost‐effective alternative for clinical settings.

Cystatin C and creatinine, as key blood biomarkers, provide a comprehensive reflection of the physiological decline across multiple systems during the ageing process. Cystatin C, a cysteine protease inhibitor, is primarily used to assess kidney function, with its levels increasing as the glomerular filtration rate (GFR) declines [24, 25, 26, 27]. As kidney function deteriorates with age, elevated cystatin C levels not only reflect renal health but are also closely associated with heightened systemic inflammation and oxidative stress [9, 28]. These physiological changes can affect multiple systems, accelerating the development of frailty by promoting cardiovascular diseases, metabolic syndrome and other chronic conditions [28, 29, 30]. At the same time, creatinine, a by‐product of muscle metabolism, reflects muscle mass [31, 32, 33, 34]. With ageing, muscle synthesis decreases and degradation increases, leading to a reduction in muscle mass and, consequently, lower creatinine levels [31]. This reduction directly contributes to the decline in physical function, particularly in terms of mobility and daily activities (such as walking, standing up and lifting objects) [34]. Many of the items in the frailty index, including chronic disease, daily activity limitations, cognitive ability and emotional health, are linked to the decline of these physiological systems [15]. The combination of cystatin C and creatinine provides a more integrated view of both renal function and muscle mass, offering a better reflection of overall physiological decline. Their ability to predict frailty and all‐cause mortality arises from their involvement in both chronic disease progression and physical function deterioration. This is where the Cystatin C‐Creatinine Score comes in. First, this score reveals the critical point of renal function through the threshold effect of renal function compensation. When the GFR drops to a critical value, the sensitive increase of cystatin C and the abnormal muscle metabolism of creatinine jointly breach the compensation threshold. This causes the retention of metabolic waste and electrolyte imbalance, accelerating the pathological process [35]. Second, an abnormal score exposes the synergistic damage between the muscle–kidney axis imbalance and inflammatory oxidative stress. Sarcopenia and renal insufficiency create a vicious cycle. Meanwhile, the build‐up of uraemic toxins activates the NLRP3 inflammasome, worsening chronic inflammation and oxidative stress, and further harming organ reserves [36]. Finally, the score initiates the non‐linear activation of ageing‐related pathways. It does this by inhibiting the mTOR pathway to reduce muscle synthesis and down‐regulating the anti‐ageing factor Klotho [37]. This non‐linear feature means the score has higher predictive power near the critical GFR value. Its combined strength lies in simultaneously capturing the interactive damage between renal function and muscle metabolism. In conclusion, the Cystatin C‐Creatinine Score, by assessing declines in the renal and muscular systems, serves as a comprehensive tool for evaluating frailty across multiple systems. It provides more accurate predictions of frailty and mortality compared to single biomarkers or epigenetic clocks.

This study presents several notable strengths. First, the use of two large, nationally representative datasets ensures broad generalizability across diverse populations, including different ethnicities, ages and health statuses. Second, the innovative integration of LASSO regression for biomarker selection and nomogram construction identifies the most parsimonious model. Unlike previous studies relying on single biomarkers or complex epigenetic clocks, the Cystatin C‐Creatinine Score combines two readily available clinical parameters, offering a cost‐effective and scalable solution for routine clinical use. Finally, the development of an open‐source web calculator bridges the translational gap, enabling clinicians to rapidly assess frailty risk during routine blood workups, thus facilitating early intervention strategies. Collectively, these strengths position the Cystatin C‐Creatinine Score as a valuable tool for precision geriatric care. This study has several limitations. First, the analysis was based on data from a single source, the HRS, which may limit the generalizability of our findings to other populations. Future research should validate the Cystatin C‐Creatinine Score in more diverse cohorts, including different ethnicities and regions. Second, although the score demonstrated strong predictive performance, only a limited range of biomarkers was considered. Third, mortality data partially relied on proxy reports, which could introduce inaccuracies, and the absence of cause‐specific mortality data limits our ability to evaluate predictions for specific death causes. Fourth, frailty was measured at a single time point, preventing us from assessing changes in frailty status over time. Longitudinal studies are needed to better understand the score's relationship with frailty progression or improvement. Fifth, despite adjusting for multiple confounders, other factors, such as diet, physical activity and medication use, may still influence the results. Additionally, whereas LASSO regression was used to select relevant biomarkers and minimize overfitting, further validation in independent cohorts is necessary to confirm the nomogram's robustness. Lastly, although the Cystatin C‐Creatinine Score offers a simpler alternative to complex epigenetic clocks, its integration into clinical practice, including cost‐effectiveness and ease of use, needs further exploration.

## 5 Conclusion

Overall, our findings contribute to the growing body of evidence supporting the utility of the Cystatin C‐Creatinine score in predicting frailty and mortality, highlighting its potential application in routine clinical practice for early detection of frailty and improved prognostic assessments in ageing populations.

## Conflicts of Interest

The authors declare no conflicts of interest.

## Supporting information


**Figure S1:** Flow chart.
**Figure S2:** Nomograms constructed using top haematological indicators selected by LASSO and corresponding ROC curve comparisons.
**Figure S3:** ROC curves of Cystatin C‐Creatinine Score for predicting frailty by 10‐fold cross‐validation.
**Figure S4:** ROC curves of Cystatin C‐Creatinine Score, Ccr and eGFR for predicting frailty.
**Figure S5:** The nonlinear relationship of Cystatin C‐Creatinine Score with frailty index and all‐cause mortality.
**Figure S6:** Correlation coefficients between epigenetic clocks, Cystatin C‐Creatinine Score, and frailty score.
**Figure S7:** Calibration curve and decision curve for predicting frailty using Cystatin C‐Creatinine Score.
**Figure S8:** Correlation between Cystatin C‐Creatinine Score and inflammation.
**Figure S9:** ROC curves and Time‐AUC of Cystatin C‐Creatinine Score for predicting frailty and all‐cause mortality.
**Figure S10:** ROC curves of Cystatin C‐Creatinine Score for predicting frailty in different subgroups.
**Figure S11:** Time‐dependent ROC curves of Cystatin C‐Creatinine Score for predicting all‐cause mortality in different subgroups.
**Table S1:** Coefficients of haematological indicators selected by LASSO regression for frailty prediction.
**Table S2:** The relationship between the Cystatin C‐Creatinine Score and functional ageing (frailty index).
**Table S3:** The relationship between the Cystatin C‐Creatinine Score and all‐cause mortality.
**Table S4:** C‐Index values and 95% confidence intervals for epigenetic clocks and Cystatin C‐Creatinine Score in predicting all‐cause mortality.

## Data Availability

The HRS datasets are publicly available. Researchers may obtain the datasets after sending a data user agreement to the HRS team (https://hrs.isr.umich.edu/data‐products).
