# Supplementary material for: The Predictive Power of the Cystatin C‐Creatinine Score in Assessing Frailty
Source: J Cachexia Sarcopenia Muscle. 2025 Aug 15;16(4):e70040. doi: 10.1002/jcsm.70040 (PMC12356705; doi:10.1002/jcsm.70040)
Supplement: Supplementary file 1 — Figure S1: Flow chart. Figure S2: Nomograms constructed using top haematological indicators selected by LASSO and corresponding ROC curve comparisons. Figure S3: ROC curves of Cystatin C‐Creatinine Score for predicting frailty by 10‐fold cross‐validation. Figure S4: ROC curves of Cystatin C‐Creatinine Score, Ccr and eGFR for predicting frailty. Figure S5: The nonlinear relationship of Cystatin C‐Creatinine Score with frailty index and all‐cause mortality. Figure S6: Correlation coefficients between epigenetic clocks, Cystatin C‐Creatinine Score, and frailty score. Figure S7: Calibration curve and decision curve for predicting frailty using Cystatin C‐Creatinine Score. Figure S8: Correlation between Cystatin C‐Creatinine Score and inflammation. Figure S9: ROC curves and Time‐AUC of Cystatin C‐Creatinine Score for predicting frailty and all‐cause mortality. Figure S10: ROC curves of Cystatin C‐Creatinine Score for predicting frailty in different subgroups. Figure S11: Time‐dependent ROC curves of Cystatin C‐Creatinine Score for predicting all‐cause mortality in different subgroups. Table S1: Coefficients of haematological indicators selected by LASSO regression for frailty prediction. Table S2: The relationship between the Cystatin C‐Creatinine Score and functional ageing (frailty index). Table S3: The relationship between the Cystatin C‐Creatinine Score and all‐cause mortality. Table S4: C‐Index values and 95% confidence intervals for epigenetic clocks and Cystatin C‐Creatinine Score in predicting all‐cause mortality. [file JCSM-16-e70040-s001.docx]

**Supplementary Methods:**

1.The Frailty Index (FI) is a multi-dimensional assessment tool used to quantify frailty in older adults. It typically includes a combination of clinical, functional, and biological markers reflecting cumulative deficits across various domains. Common components of a Frailty Index may include: **Physical Function:** Grip strength, Walking speed, Balance/standing tests, Activities of daily living (ADL) dependence; **Nutritional Status:** Weight loss, Low body mass index (BMI), Serum albumin levels; **Cognitive/Psychological Factors:** Cognitive impairment (e.g., Mini-Mental State Examination, MMSE), Depressive symptoms (e.g., Geriatric Depression Scale, GDS); **Comorbidities:** Number of chronic diseases (e.g., hypertension, diabetes, arthritis), Disease severity or complications; **Social/Environmental Factors:** Social isolation, Limited social support, Functional dependence (e.g., instrumental ADL, IADL); **Other Biomarkers:** Inflammatory markers (e.g., IL-6, CRP), Anemia (low hemoglobin) , Low muscle mass (e.g., via DXA scans, bioelectrical impedance).

2. We included 40 indicators, including: cystatin c (mg/l), creatinine (mg/dl), albumin (g/dl), bilirubin, total (mg/dl), red cell distribution width (%), hemoglobin (g/dl), red blood cell count (10^12^/l), dehydroepiandrosterone sulfate (umol/l), mean corpuscular hemoglobin concentration (g/dl), chloride (mmol/l), glucose, fasting (mg/dl), cholesterol, total (mg/dl), alkaline phosphatase (U/L), c-reactive protein (high sensitivity) (mg/l), hdl-cholesterol, direct-measure (mg/dl), b-type natriuretic peptide, b-terminal pro (nt-proBNP) (pg/mL), eosinophil count (10^9^/l), bicarbonate (HCO₃⁻) (mmol/l), urea nitrogen (BUN) (mg/dl), platelet distribution width (fl), potassium (mmol/l), sodium (mmol/l), lymphocyte count (10^9^/l), alanine aminotransferase (U/L), platelet count (10^9^/l), aspartate aminotransferase (U/L), ferritin (μg/l), CMV IgG (COI), basophil count (10^9^/l), monocyte count (10^9^/l), neutrophil count (10^9^/l), percent basophils (%), calcium (mg/dl), percent eosinophils (%), hematocrit (%), ldl-cholesterol, calculated (mg/dl), percent lymphocytes (%), mean corpuscular hemoglobin (pg), mean corpuscular volume (fl), percent monocytes (%), mean platelet volume (fl), percent neutrophils (%), triglycerides (mg/dl), protein, total (g/dl), white blood cell count (10^9^/l). Subsequently, the Least Absolute Shrinkage and Selection Operator (LASSO) regression model was used to screen these variables. LASSO regression is a statistical method for high-dimensional data analysis that introduces an L1 regularization constraint, enabling the regression model to automatically select the variables most influential in predicting the degree of frailty. After identifying the key hematological biomarkers through LASSO regression, this study constructed a nomogram based on these variables. Considering the large number of included factors, we then compared the accuracy of the model when different numbers of variables were included. Finally, it was determined that only creatinine and cystatin C played a major role. Based on this result, a web calculator was constructed in shiny app, mainly including two factors: blood creatinine level and cystatin C level.

3.The "maxstat" method automatically optimizes the cutoff value through statistical models, avoiding the limitations of subjective empirical judgments (such as manually setting thresholds or using medians). It is particularly suitable for binary classification problems (e.g., frailty prediction) based on biomarkers. This method determines the optimal segmentation point by maximizing specific statistical metrics (such as the Youden index, likelihood ratio, or predictive performance indicators), ensuring that the cutoff value is highly consistent with the characteristics of the data distribution[1].

[1]Hothorn, T. and Lausen, B. (2003). On the Exact Distribution of Maximally Selected Rank Statistics. Computational Statistics & Data Analysis, 43, 121–137.

**Figure S1. Flow chart**

**
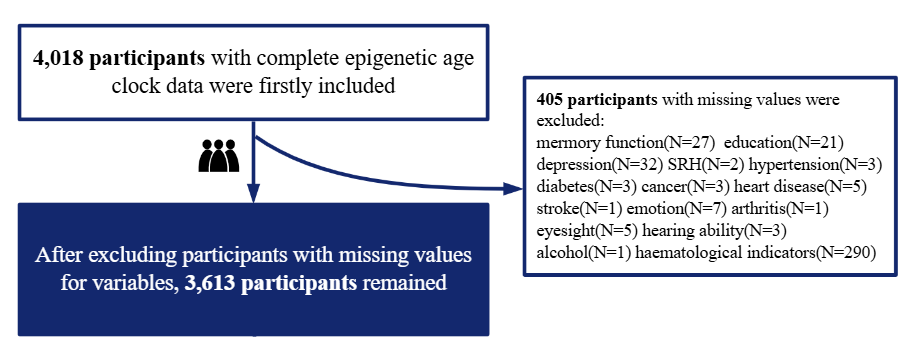
**

**
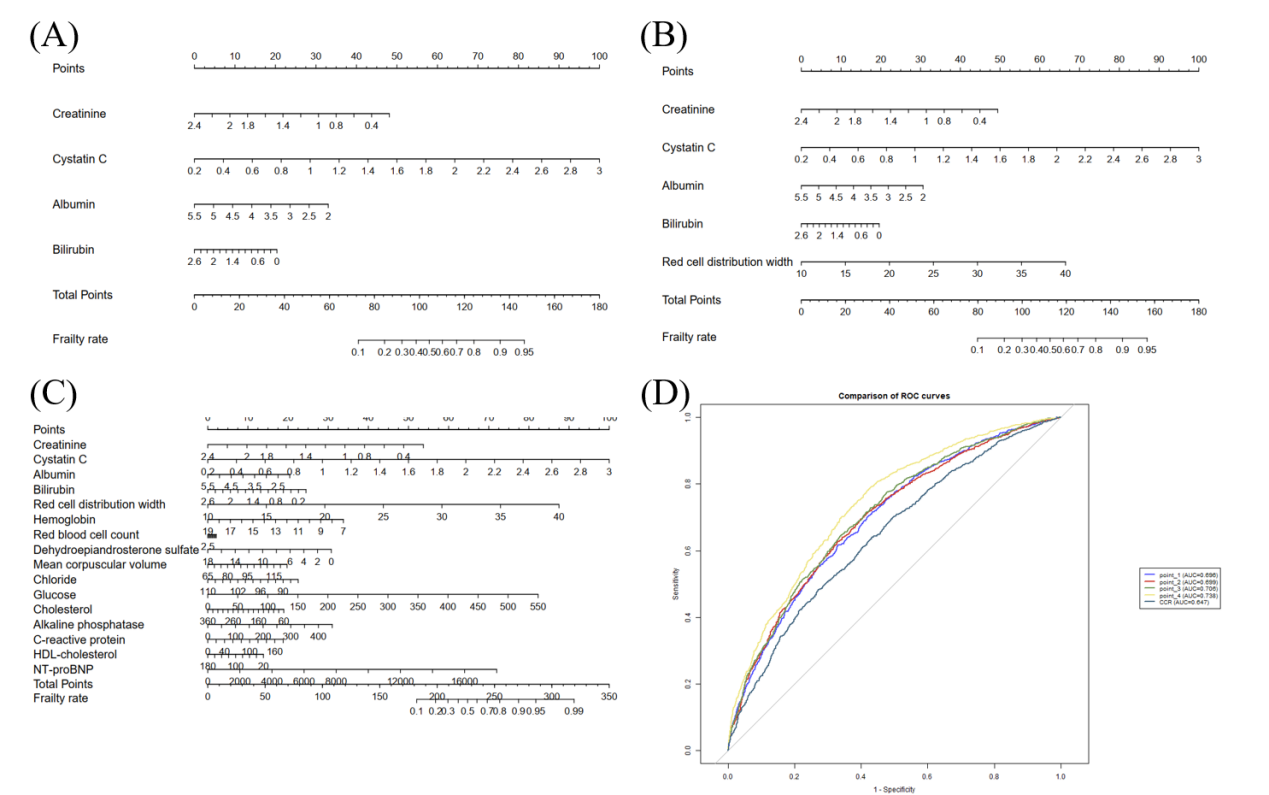
Figure S2. Nomograms constructed using top hematological indicators selected by LASSO and corresponding ROC curve comparisons**

**Notes:** (A) Nomogram using Top 4 hematological indicators for frailty prediction; (B) Nomogram using Top 5 hematological indicators for frailty prediction; (C) Nomogram using Top 16 hematological indicators for frailty prediction; (D) ROC curve comparison of nomograms with different numbers of hematological indicators; point_1: Nomogram constructed using creatinine and cystatin C; point_2: Nomogram score from Figure S2A, using the top 4 hematological indicators; point_3: Nomogram score from Figure S2B, using the top 5 hematological indicators; point_4: Nomogram score from Figure S2C, using the top 16 hematological indicators; CCR, Creatinine to Cystatin C Ratio

**Figure S3. ROC curves of Cystatin C-Creatinine Score for predicting frailty by 10-fold cross-validation**

**
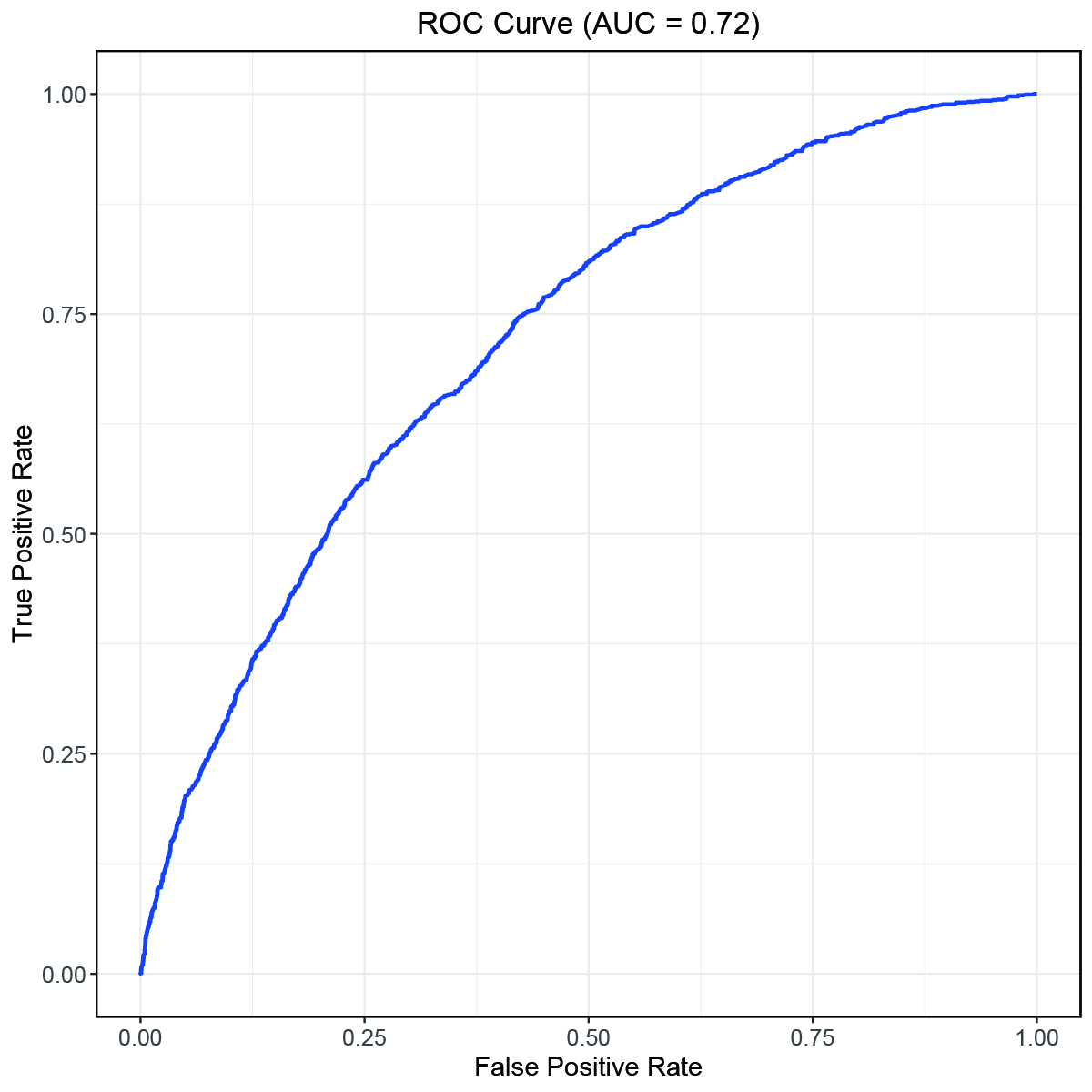
**

**Figure S4. ROC curves of Cystatin C-Creatinine Score, Ccr, and eGFR for predicting frailty**


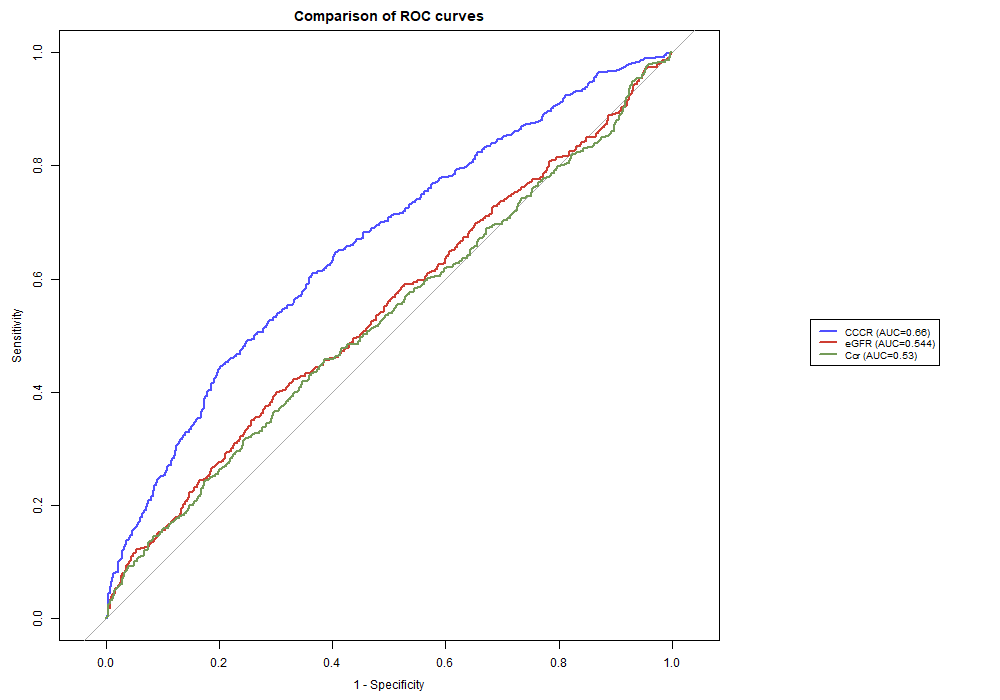


**
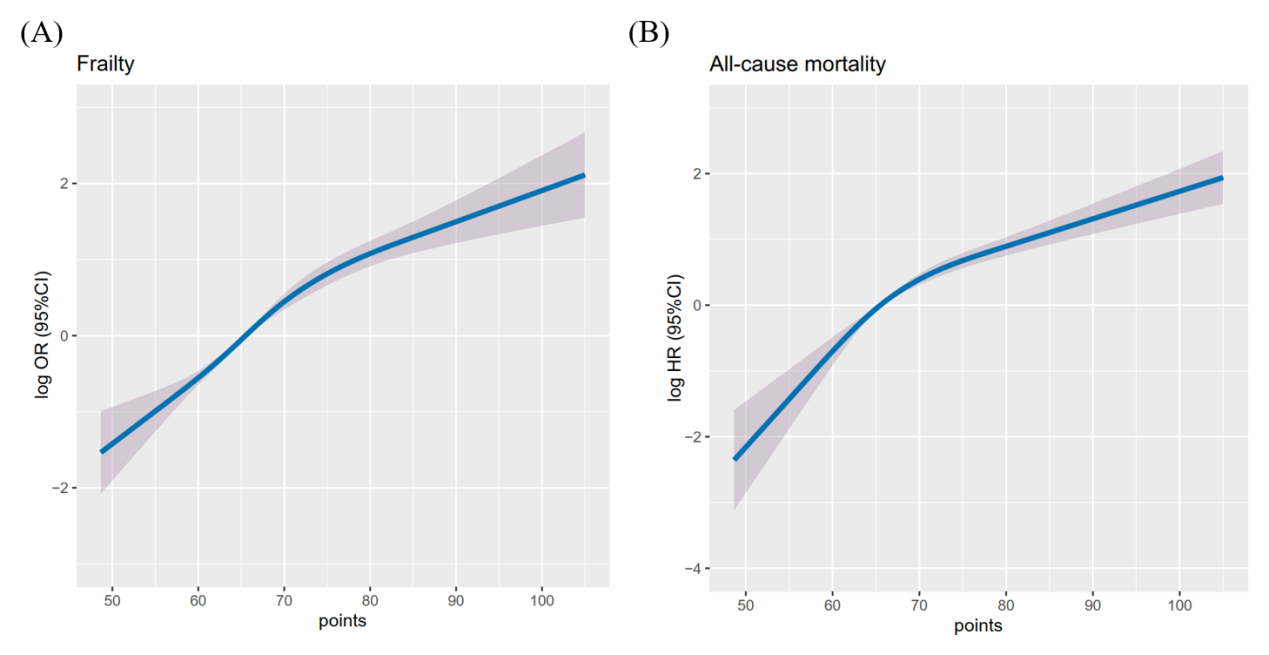
Figure S5. The nonlinear relationship of Cystatin C-Creatinine Score with frailty index and all-cause mortality**

**Notes:**(A) shows the nonlinear relationship between Cystatin C-Creatinine Score and frailty index; (B) shows the nonlinear relationship between Cystatin C-Creatinine Score and all-cause mortality.

**
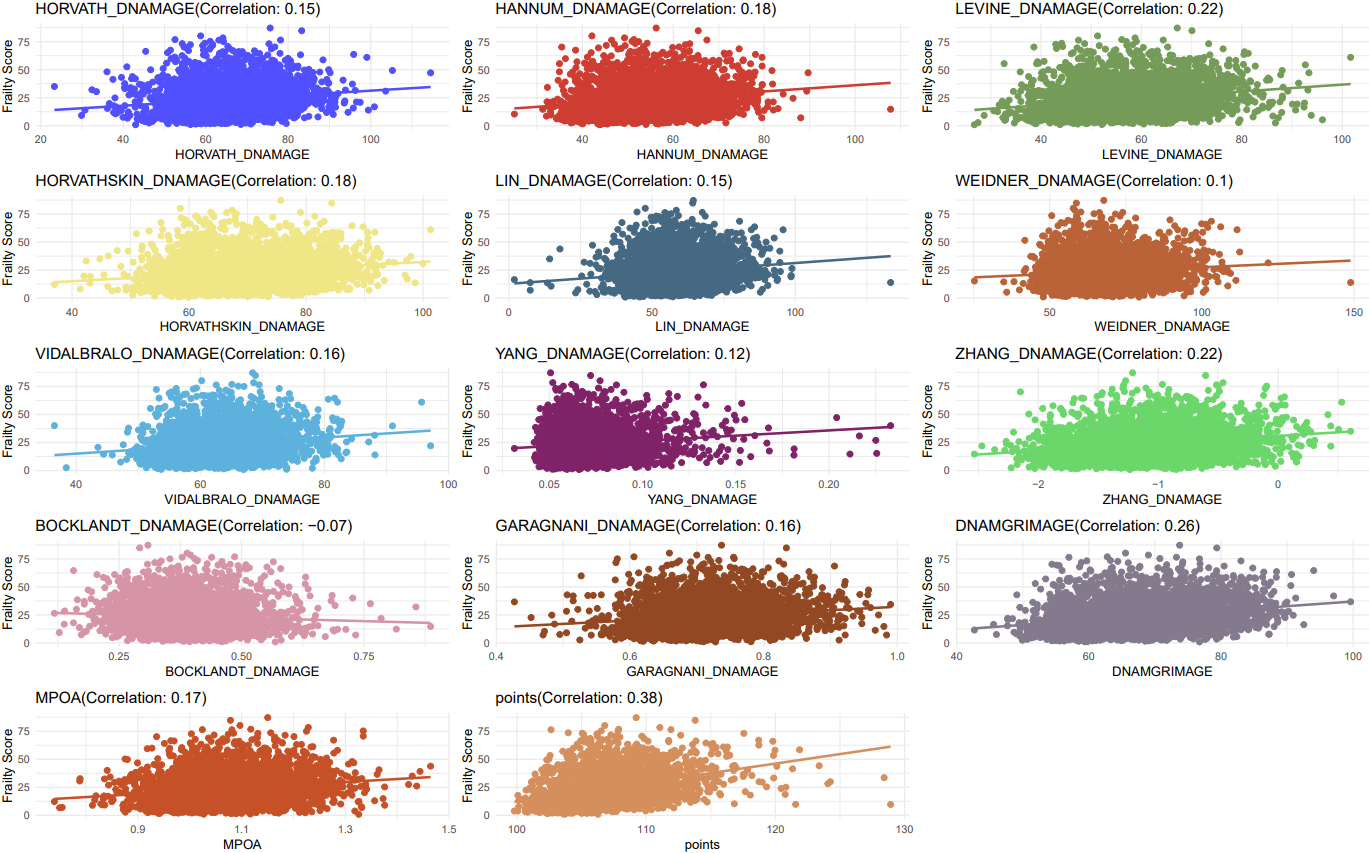
Figure S6. Correlation coefficients between epigenetic clocks, Cystatin C-Creatinine Score, and frailty score**

**
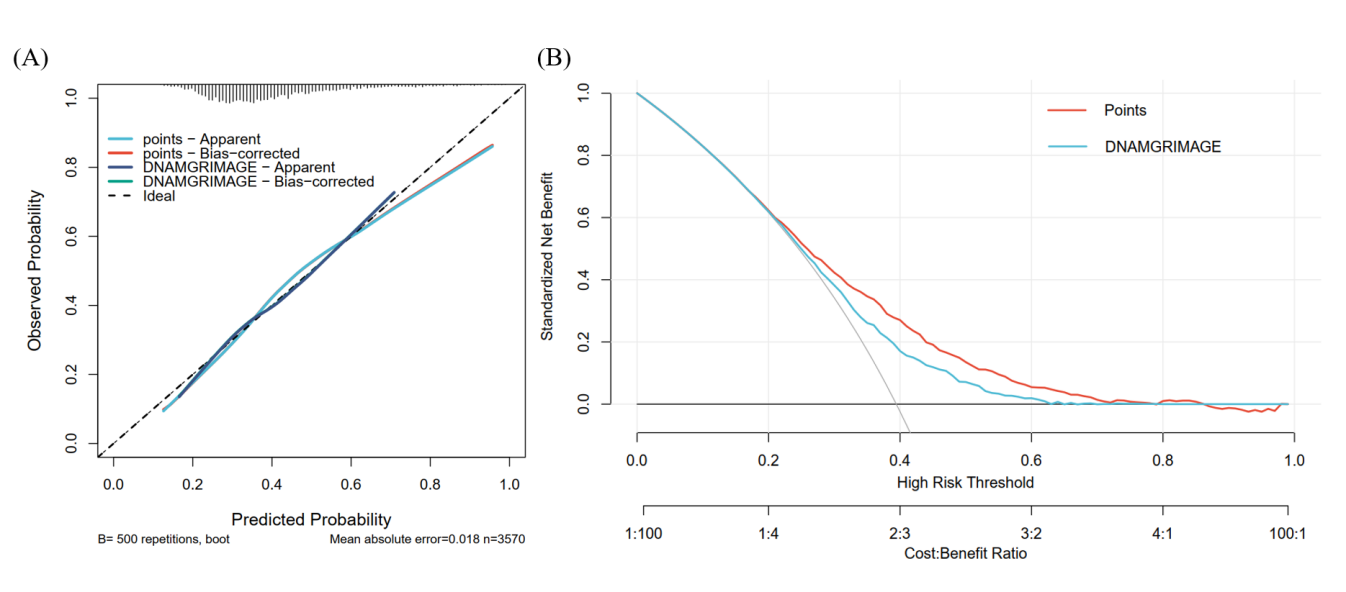
Figure S7. Calibration curve and decision curve for predicting frailty using Cystatin C-Creatinine Score**

**Notes:** (A) Calibration curve comparing the predicted probability and observed probability for frailty using the Cystatin C-Creatinine Score and DNAmGrimAge; (B) Decision curve analysis for frailty prediction using the Cystatin C-Creatinine Score and DNAmGrimAge, illustrating net benefit across different risk thresholds.

**Figure S8. Correlation between Cystatin C-Creatinine Score and inflammation**

**
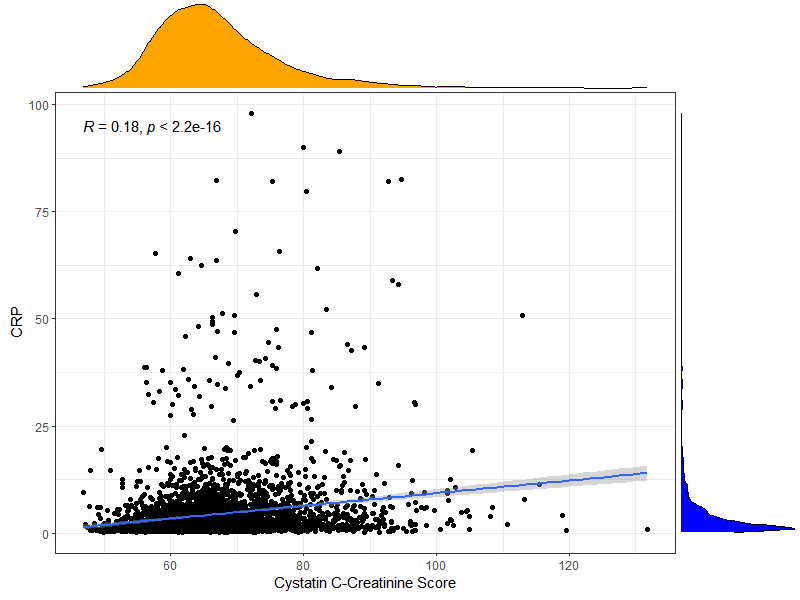
**

**Figure S9. ROC curves and Time-AUC of Cystatin C-Creatinine Score for predicting frailty and all-cause mortality**

**
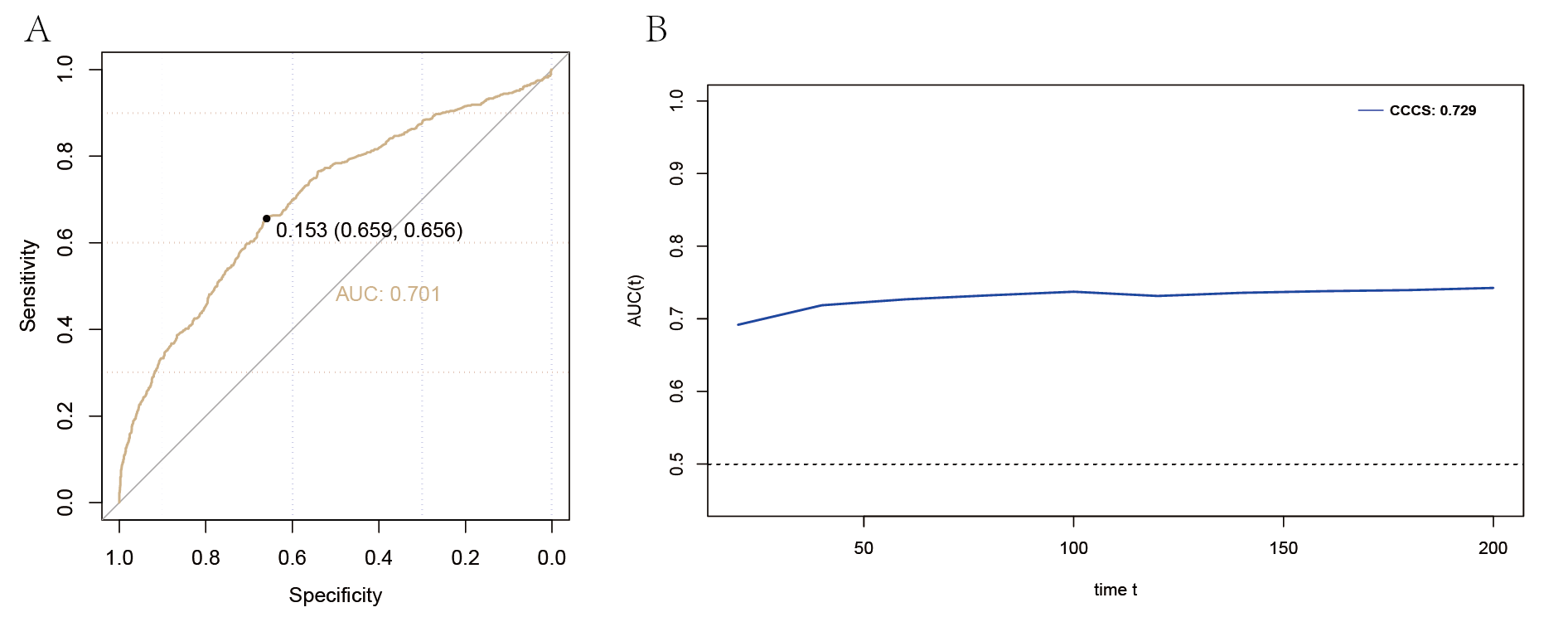
**

**Notes:** (A) ROC curves of Cystatin C-Creatinine Score for predicting frailty; (B) Time-AUC of Cystatin C-Creatinine Score for predicting all-cause mortality

**Figure S10. ROC curves of Cystatin C-Creatinine Score for predicting frailty in different subgroups**

**Notes:** (A) Participants aged < 70 years; (B) Participants aged ≥ 70 years; (C) Male participants;(D) Female participants; (E) Unmarried participants; (F) Married participants;(G) Participants with low educational attainment; (H) Participants with high educational attainment; (I) Participants without hypertension; (J) Participants with hypertension; (K) Participants without diabetes; (L) Participants with diabetes; (M) Participants without cancer; (N) Participants with cancer.

**Figure S11. Time-dependent ROC curves of Cystatin C-Creatinine Score for predicting all-cause mortality in different subgroups**

**Notes:** (A) Participants aged < 70 years; (B) Participants aged ≥ 70 years; (C) Male participants;(D) Female participants; (E) Unmarried participants; (F) Married participants;(G) Participants with low educational attainment; (H) Participants with high educational attainment; (I) Participants without hypertension; (J) Participants with hypertension; (K) Participants without diabetes; (L) Participants with diabetes; (M) Participants without cancer; (N) Participants with cancer.

**Table S1: Coefficients of hematological indicators selected by LASSO regression for frailty prediction**

| **Variable** | **Coefficient^*^** | **Coefficient^#^** |
| --- | --- | --- |
| cystatin c - mg/l | 1.631137E+00 | 1.267521E+00 |
| creatinine - mg/dl | -7.789560E-01 | -3.604153E-01 |
| albumin - g/dl | -2.507696E-01 | -3.087372E-01 |
| bilirubin, total - mg/dl | -3.974043E-01 | -1.630372E-01 |
| red cell distribution width - % | 1.253111E-01 | 8.614167E-02 |
| hemoglobin - g/dl | -2.100408E-02 | -7.990480E-02 |
| red blood cell count - 10e12/l | -2.978258E-01 | -6.963109E-02 |
| dehydroepiandrosterone sulfate (dheas) - umol/l | -6.970054E-02 | -5.904387E-02 |
| mean corpuscular hemoglobin concentration - g/dl | -4.297582E-02 | -3.594411E-02 |
| chloride - mmol/l | -4.015648E-02 | -2.889045E-02 |
| glucose, fasting - mg/dl | 7.065044E-03 | 6.081298E-03 |
| cholesterol, total - mg/dl | -2.687802E-03 | -1.981272E-03 |
| alkaline phosphatase - U/L | 2.808515E-03 | 1.933903E-03 |
| c-reactive protein (high sensitivity) - mg/l | 4.779789E-03 | 1.630701E-03 |
| hdl-cholesterol, direct-measure - mg/dl | -2.656720E-03 | -2.974416E-04 |
| b-type natriuretic peptide, b-terminal pro (nt-probnp)-pg/mL | 1.442056E-04 | 7.315394E-05 |
| eosinophil count - x10e9/l | 9.267216E-02 | None |
| bicarbonate (co2) - mmol/l | 1.236434E-02 | None |
| urea nitrogen (bun) - mg/dl | -1.232396E-02 | None |
| platelet distribution width - fl | 1.065205E-02 | None |
| potassium - mmol/l | -9.498220E-03 | None |
| sodium - mmol/l | 3.441150E-03 | None |
| lymphocyte count - x10e9/l | -8.405932E-04 | None |
| alanine aminotransferase - U/L | -5.915886E-04 | None |
| platelet count - 10e9/l | -2.981005E-04 | None |
| aspartate aminotransferase - U/L | -2.933419E-04 | None |
| ferritin - ug/l | -2.596366E-04 | None |
| cmv igg - coi | 1.838996E-05 | None |
| basophil count - x10e9/l | None | None |
| monocyte count - x10e9/l | None | None |
| neutrophil count - x10e9/l | None | None |
| percent basophils - % | None | None |
| calcium - mg/dl | None | None |
| percent eosinophils - % | None | None |
| hematocrit - % | None | None |
| ldl-cholesterol, calculated - mg/dl | None | None |
| percent lymphocytes - % | None | None |
| mean corpuscular hemoglobin - pg | None | None |
| mean corpuscular volume - fl | None | None |
| percent monocytes - % | None | None |
| mean platelet volume - fl | None | None |
| percent neutrophils - % | None | None |
| triglycerides - mg/dl | None | None |
| protein, total - g/dl | None | None |
| white blood cell count - 10e9/l | None | None |

**Notes:** * Coefficients derived using the lambda value that minimizes prediction error, potentially selecting more variables; # Right column: Coefficients derived using the lambda value with 1-standard error, resulting in a more regularized model with fewer variables.

**Table S2. The relationship between the Cystatin C-Creatinine Score and functional aging (frailty index)**

|  | **frailty/participants** | **Model 1** | **P_value** | **Model 2** | **P_value** | **Model 3** | **P_value** |
| --- | --- | --- | --- | --- | --- | --- | --- |
| **As continuous** | 1410/3570 | 1.02 (1.02,1.02) | <0.001 | 1.01 (1.01,1.02) | <0.001 | 1.01 (1.01,1.01) | <0.001 |
| **By cutoff** |  |  |  |  |  |  |  |
| **< 67.7** | 620/2168 | ref | ref | ref | ref | ref | ref |
| **≥ 67.7** | 790/1402 | 1.32 (1.28,1.36) | <0.001 | 1.24 (1.20,1.29) | <0.001 | 1.20 (1.16,1.24) | <0.001 |
| **By quartile** |  |  |  |  |  |  |  |
| **Q1 (~60.85)** | 190/899 | ref | ref | ref | ref | ref | ref |
| **Q2 (60.85~65.55)** | 275/886 | 1.10 (1.06,1.15) | <0.001 | 1.09 (1.04,1.14) | <0.001 | 1.08 (1.04,1.13) | <0.001 |
| **Q3 (65.55~71.56)** | 398/892 | 1.26 (1.21,1.32) | <0.001 | 1.21 (1.16,1.27) | <0.001 | 1.19 (1.13,1.24) | <0.001 |
| **Q4 (71.56~)** | 547/893 | 1.49 (1.43,1.56) | <0.001 | 1.39 (1.33,1.46) | <0.001 | 1.33 (1.27,1.40) | <0.001 |
| **P for trend** |  |  | <0.001 |  | <0.001 |  | <0.001 |

Model 1: no adjusted

Model 2: adjusted for age, sex, marital status and education level

Model 3: adjusted for age, sex, marital status, education level, smoking status, alcohol consumption, albumin, bilirubin, alanine aminotransferase and alkaline phosphatase

**Table S3. The relationship between the Cystatin C-Creatinine Score and all-cause mortality**

|  | **death/participants** | **Model 1** | **P_value** | **Model 2** | **P_value** | **Model 3** | **P_value** |
| --- | --- | --- | --- | --- | --- | --- | --- |
| **As continuous** | 307/3570 | 1.06 (1.05,1.07) | <0.001 | 1.06 (1.05,1.07) | <0.001 | 1.03 (1.02,1.04) | <0.001 |
| **By cutoff** |  |  |  |  |  |  |  |
| **< 67.7** | 88/2168 | ref | ref | ref | ref | ref | ref |
| **≥ 67.7** | 219/1402 | 4.03 (3.14,5.16) | <0.001 | 2.33 (1.78,3.06) | <0.001 | 2.06 (1.56,2.71) | <0.001 |
| **By quartile** |  |  |  |  |  |  |  |
| **Q1 (~60.85)** | 28/899 | ref | ref | ref | ref | ref | ref |
| **Q2 (60.85~65.55)** | 30/886 | 1.05 (0.63,1.76) | 0.849 | 0.93 (0.56,1.57) | 0.794 | 0.89 (0.53,1.49) | 0.655 |
| **Q3 (65.55~71.56)** | 78/892 | 2.78 (1.81,4.29) | <0.001 | 1.84 (1.18,2.88) | 0.007 | 1.63 (1.04,2.56) | 0.034 |
| **Q4 (71.56~)** | 171/893 | 6.49 (4.35,9.68) | <0.001 | 3.19 (2.06,4.93) | <0.001 | 2.64 (1.69,4.11) | <0.001 |
| **P for trend** |  |  | <0.001 |  | <0.001 |  | <0.001 |

Model 1: no adjusted

Model 2: adjusted for age, sex, marital status and education level

Model 3: adjusted for age, sex, marital status, education level, smoking status, alcohol consumption, albumin, bilirubin, alanine aminotransferase and alkaline phosphatase

**Table S4. C-Index values and 95% confidence intervals for epigenetic clocks and Cystatin C-Creatinine Score in predicting all-cause mortality**

| **C-index (95% confidential interval)** | | | |
| --- | --- | --- | --- |
| HORVATH_DNAMAGE | 0.697 (0.664, 0.73) | YANG_DNAMAGE | 0.552 (0.518, 0.587) |
| HANNUM_DNAMAGE | 0.728 (0.699, 0.758) | ZHANG_DNAMAGE | 0.724 (0.697, 0.751) |
| LEVINE_DNAMAGE | 0.740 0.712, 0.768) | BOCKLANDT_DNAMAGE | 0.655 (0.622, 0.688) |
| HORVATHSKIN_DNAMAGE | 0.711 (0.68, 0.742) | GARAGNANI_DNAMAGE | 0.677 (0.645, 0.709) |
| LIN_DNAMAGE | 0.693 (0.661, 0.724) | DNAMGRIMAGE | 0.781 (0.756, 0.806) |
| WEIDNER_DNAMAGE | 0.619 (0.586, 0.652) | MPOA | 0.625 (0.594, 0.657) |
| VIDALBRALO_DNAMAGE | 0.689 (0.658, 0.720 | Cystatin C-Creatinine Score | 0.717 (0.688, 0.747) |
